# Antioxidant and Anti-Inflammatory Properties of Hydroxyl Safflower Yellow a in Diabetic Nephropathy: A Meta-Analysis of Randomized Controlled Trials

**DOI:** 10.3389/fphar.2022.929169

**Published:** 2022-08-11

**Authors:** Shunlian Fu, Qian Zhou, Yang Gao, Yunjiao Yang, Huizhen Chen, Lijun Yuan, Zinan Li, Qiu Chen

**Affiliations:** Hospital of Chengdu University of Traditional Chinese Medicine, Chengdu, China

**Keywords:** diabetic kidney disease, oxidative stress, hydroxyl safflower yellow A, inflammatory, meta-analysis

## Abstract

**Background:** Diabetic kidney disease (DKD) is a chronic progressive disorder which is a leading cause of chronic kidney disease (CKD). As an important pathogenesis of DKD, the overproduction of reactive oxygen species (ROS) and the inflammatory response have been considered central mediators in the progression of DKD. Herbal products are increasingly being applied as antioxidants and anti-inflammatory agents. Of those, the effect of hydroxyl safflower yellow A (HSYA) on oxidative stress and inflammatory reactions has gradually been investigated for DKD treatment, which may provide therapies for DKD with new insights and promote its application in clinical practice.

**Methods:** We searched CNKI, the Chinese Biomedical Literature Database, the Wanfang Database, PubMed, and Embase from the establishment date of the database to 22 April 2022. The included literature in our study was randomized controlled trials (RCTs) using HSYA to treat DKD. We performed a meta-analysis by calculating the standard mean difference (SMD) with a 95% confidence interval (CI). The inverse-variance method with a random effect was used in our meta-analysis using Stata software and RevMan software.

**Results:** A total of 31 articles with 31 groups containing a total of 2487 participants were included in this meta-analysis. The pooled results showed a statistical improvement in the following measurements: fasting blood glucose (FBG), postprandial blood glucose (PBG), blood urea nitrogen (BUN), urinary albumin excretion rates (UAER), serum creatinine (SCR), hypersensitive C-reactive protein (hsCRP), interleukin-6 (IL-6), tumor necrosis factor-α (TNF-α), fasting insulin (FINS), total cholesterol (TC), triglycerides (TGs), hemoglobin A1c (HbA1C), homeostasis model assessment insulin resistance (HOMA-IR), and malondialdehyde (MDA).

**Conclusion:** HSYA can effectively treat DKD by inhibiting inflammatory reactions and oxidative stress, decreasing blood glucose and blood lipids, and improving renal function indices. However, more RCTs are still needed in the future to further demonstrate the effect of HSYA on biomarkers of oxidative stress and inflammatory reactions in patients with DKD due to the low quality and small sample size of the literature included in this study.

**Systematic Review Registration:** PROSPERO: CRD 42021235689

## Introduction

Of the long-term complications of diabetes, diabetic kidney disease (DKD) imposes the highest burden in terms of financial cost and daily life effects, leading to increasing cases of end-stage renal disease (ESRD) (Mottl et al., 2022; Thomas et al., 2015). Current Western medicine (WM) treatment for DKD focuses on the intense control of glycemic levels and blood pressure and is inadequate at postponing an apparent progression to end-stage renal disease (ESRD) in patients (Fernandez-Fernandez et al., 2014), forcing us to explore more effective treatments and further optimize protection in DKD.

Safflower (*Carthamus tinctorius* L.), a traditional Chinese medicine plant, is widely used to improve cardiovascular and cerebrovascular diseases in China and for scavenging oxygen-free radicals, reducing inflammatory infiltration, and inhibiting inflammatory apoptosis (Xue et al., 2021). Safflower yellow is the main active component extracted from safflower, containing safflower yellow A, safflower yellow B, and hydroxyl safflower yellow A (HSYA) (Xue et al., 2021; Qi et al., 2014). HSYA is the major bioactive index component among these components, accounting for 85% of safflower yellow (Fangma et al., 2021). HSYA has been commonly used in treating cardiovascular and cerebrovascular diseases in China and to scavenge oxygen-free radicals, reduce inflammatory infiltration, and inhibit apoptosis (Xue et al., 2021). Recently, several reviews and meta-analyses (SRs/MAs) have indicated that HSYA can significantly improve DKD by enhancing the total effective rate and reducing urinary albumin excretion rates (UAERs), serum creatinine (SCR), and blood urea nitrogen (BUN) levels (Yang et al., 2018; Wang et al., 2019a; Wang et al., 2019b; Jin et al., 2019; Xu et al., 2019). However, the underlying mechanism implicated in the role of HSYA in DKD remains unclear. In some human trials, some randomized controlled trials (RCTs) have discussed the therapeutic effect of HSYA on oxidative stress and the inflammatory response in patients with DKD (Yin, 2018; Zhang, 2018). However, there are some controversies about the therapeutic effect of HSYA on biomarkers of oxidative stress.

Additionally, multiple factors, including small sample sizes, the duration of intervention, and small study effects, can often affect the reliability of conclusions about the antioxidant or anti-inflammatory properties of HSYA in treating DKD. Moreover, the adverse effects of HSYA in managing patients with DKD remain unclear. Furthermore, whether the methodological quality and publication bias may overstate the efficacy of HSYA is still uncertain.

Therefore, this meta-analysis aimed to assess existing RCTs using HSYA to treat patients with DKD, clarifying the effect of HSYA on oxidative stress and inflammatory cytokines in patients with DKD. The effect of HSYA on indices related to renal function, blood glucose, and lipids was further assessed in this study. Additionally, the pathogenesis of DKD from the ROS and inflammatory response perspective was further elucidated in this study. The multitargeted antioxidative stress and anti-inflammatory properties of HSYA provide valuable references and implications for alternative therapies and promote the application of HSYA in clinical practice.

## Methods

We performed this meta-analysis by following the guidelines in the Cochrane handbook, preferred reporting items for systematic reviews and meta-analyses protocols (PRISMA-P) (Moher et al., 2016), and PRISMA (Page et al., 2021) (see Supplementary Table S3). Our study was registered in PROSPERO (CRD 42021235689). The PRISMA-ScR checklist was utilized to check the study.

### Search Strategies

In this study, RCTs on the effect of HSYA combined with WM on antioxidant and anti-inflammatory properties in patients with DKD were searched for in the CNKI, Chinese Biomedical Literature Database, Wanfang Database, PubMed, and Embase databases. The included literature was only in Chinese or English, and the retrieval date was from the establishment date of the database to 22 April 2022. We also searched all ongoing RCTs on related websites, such as ISRCTN (the International Standard Randomized Controlled Trial Number register). The keywords were as follows: (“hydroxyl safflower yellow A” or “hydroxyl safflower yellow pigment A” or “safflower yellow”) and (“nephropathies, diabetic” or “nephropathy, diabetic” or “diabetic nephropathy” or “diabetic kidney disease” or “diabetic kidney diseases” or “kidney disease, diabetic” or “kidney diseases, diabetic” or “diabetic glomerulosclerosis” or “glomerulosclerosis, diabetic” or “intracapillary glomerulosclerosis” or “nodular glomerulosclerosis” or “glomerulosclerosis, nodular”). In addition, references were manually searched to avoid missing relevant literature studies. The related literature was imported into document management software (EndNote version X9).

### Inclusion and Exclusion Criteria

We checked each article by following predefined criteria. The literature inclusion criteria were as follows: 1) participants: the included participants were patients with DKD; 2) interventions: based on the treatment of the control group, HSYA was given intravenously, and the dose, frequency, or duration was not restricted; 3) comparison: the comparison was set as Western medicine, other conventional treatments, Western medicine combined with other conventional treatments, or other traditional Chinese medicine injections; 4) outcomes: the primary outcomes were measurement fasting blood glucose (FBG), serum creatinine (SCR), postprandial blood glucose (PBG), UAER, and blood urea nitrogen (BUN) levels. The secondary outcomes were indices related to antioxidant and anti-inflammatory properties, which were mainly superoxide dismutase (SOD), glutathione peroxidase (GSH-Px), malondialdehyde (MDA), nuclear factor-κB (NF-κB), interleukin-10 (IL-10), interleukin-6 (IL-6), tumor necrosis factor-α (TNF-α), interleukin-1β (IL-1β), and hypersensitive C-reactive protein (hsCRP). Other outcomes were biochemical indices, including fasting insulin (FINS), homeostasis model assessment of insulin resistance (HOMA-IR), adverse reactions, triglycerides (TGs), total cholesterol (TC), and hemoglobin A1c (HbA1c) levels.

The literature exclusion criteria were as follows: the research subject was non-DKD, and the research objects were cells or animal models, conference articles, letters, commentary articles, editorials, and studies where complete data could not be obtained from the literature by contacting the corresponding authors; HSYA was administered by nonvenous means. We only included RCTs published in English and Chinese, whereas non-RCTs, such as reviews, meta-analyses, or case reports, were excluded.

### Study Selection

We searched the literature by reference to the predefined keywords. The retrieved literature was imported into EndNote X9 software, and then, by reading titles and abstracts, two researchers independently screened the literature. Finally, the full text was checked to ensure that it met the inclusion criteria. Any difference between the two researchers was further resolved by discussion with a third researcher.

### Data Extraction

Two researchers independently carried out data extraction, and a third researcher checked the extracted data. The following data were extracted: the first author, the published year, the number of participants in the intervention group and the control group, the duration of intervention, the dose of HSYA, and the measured outcomes, including IL-1β, IL-6, IL-10, TNF-α, NF-κB, SOD, GSH-Px, MDA, FBG, SCR, PBG, UAER, and BUN.

### Study Quality Assessment

By using the Cochrane Collaborations tool (Higgins et al., 2011), we evaluated the study’s quality using the following seven criteria: 1) randomness of sequence generation, 2) the concealment of allocation, 3) blindness of participants and staff, 4) blindness in outcome assessment, 5) incompleteness of resulting data, 6) selective reporting bias, and 7) other possible biases. Two authors independently performed this process. A third person resolved any ambiguity or discrepancy in this course.

### Strategy for Data Analysis

When pooling the results, for all the included data that were continuous variables, the standard mean difference (SMD) with 95% confidence interval (CI) by inverse-variance with a random effect was calculated. I^2^ statistics or Cochran’s Q test was calculated to detect the magnitude of heterogeneity (Fu et al., 2022). For Cochrane’s Q test, when *p* < 0.1, we considered the results to indicate significant heterogeneity; for I^2^ statistics, when the I^2^ value was > 50%, we considered the results to indicate significant heterogeneity. The publication bias and small-study effect were estimated by Begg’s test and Egger’s test. The subgroup was taken by the dose and duration of intervention. Sensitivity analyses were performed by removing one study at a time to evaluate the robustness of the results. All statistical analyses were performed by Review Manager software (RevMan, V.5.3 for Macintosh; The Cochrane Collaboration) and Stata software version 15.1 (Stata Corporation).

## Results

### Literature Screening Results

**FIGURE 1:**
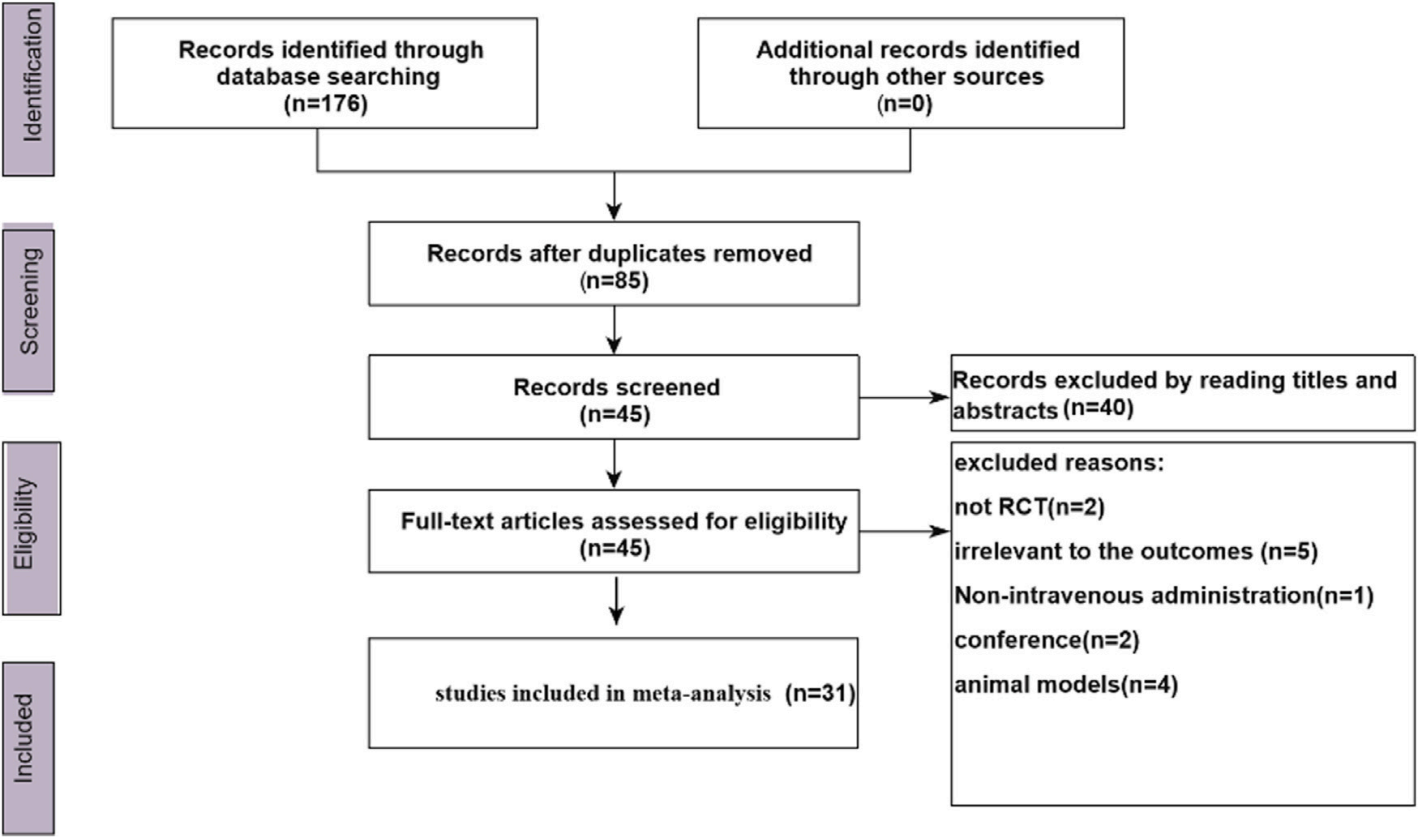
Flow chart.

A total of 176 literature items were initially obtained, and the flow chart is presented in Figure 1. After removing duplicate studies, 85 articles remained. Based on the title or abstracts, we excluded 40 articles. Forty-five articles were checked by browsing the full-text review. According to the predefined inclusion and exclusion criteria, we only selected a total of 31 articles (Yin, 2018; Zhang, 2018; Guo et al., 2008; Guo and Jiao, 2010; Yang and Yang, 2010; Zhang, 2010; Yang et al., 2011; Bai et al., 2012; Zhi and Wang, 2012; Qiu et al., 2013; Zhao and Zhao, 2013; Xu and He, 2014; Yu et al., 2014; Zhang, 2014; Gao et al., 2015a; Gao et al., 2015b; Gao et al., 2015c; Fang, 2015; Shi, 2015; Wang et al., 2016; Bao et al., 2017a; Bao et al., 2017b; Wang et al., 2017; Yang, 2017; Xie et al., 2018; Liu et al., 2019; Wu et al., 2019; Xiao and Gu, 2019; Xu and Shou, 2019; Guo, 2020; Wang et al., 2021a) for the meta-analysis. All the included articles were published in China, all the included populations were Chinese, and a total of 2487 participants were involved in this study. (See Supplementary Table S3 for details).

## Quantitative Data Analysis

### Effect of Hydroxyl Safflower Yellow A on Indicators Related to Blood Glucose and Blood Lipids

Compared with the control group, the pooled results of 14 trials showed that the HSYA group had lower FBG levels (SMD = −0.63; 95% CI: −1.05, −0.21; *p* = 0.003; P_He_ < 0.001; I^2^ = 91.8%) (Supplementary Figure S1). Eight groups evaluating PBG levels demonstrated similar results (SMD = −0.51; 95% CI: −0.98, −0.03; *p* = 0.038; P_He_ < 0.001; I^2^ = 90.7%) (Supplementary Figure S2). The HSYA group also had a better effect on HOMA-IR (SMD = −1.35; 95% CI: −2.06, −0.65; *p* < 0.001; PHe < 0.001; I^2^ = 90.8%) (Supplementary Figure S3) and on HbA1C (SMD = −0.55; 95% CI: −1.11, −0.00; *p* = 0.05; P_He_ < 0.001; I^2^ = 87.4%) (Supplementary Figure S4). Similarly, the HSYA group had lower TC (SMD = −1.03; 95% CI: −1.25, −0.80; *p* < 0.001; P_He_ < 0.001; I^2^ = 85.8%) (Supplementary Figure S5), TG levels (SMD = −1.05; 95% CI: −1.28, -0.82; *p* = 0.002; P_He_ < 0.001; I^2^ = 89.5%) (Supplementary Figure S6), and the FINS level was also lower than that of the control group (SMD = −1.58; 95% CI: −2.80, −0.36; *p* = 0.011; P_He_ < 0.001; I^2^ = 95.1%) (Supplementary Figure S7). However, one problem we cannot ignore is that the results of all the aforementioned indicators had significant heterogeneity.

### Effect of Hydroxyl Safflower Yellow A on Biomarkers of Inflammation

In this meta-analysis, the effects of HSYA on the following inflammatory biomarkers were recorded: hsCRP, IL-6, IL-10, and TNF-α. The results were pooled by using a random effects model. There was a significant decrease following HSYA administration in hsCRP (SMD = −1.96; 95% CI: −2.55, −1.38; *p* = 0.000; P_He_ < 0.001; I^2^ = 91.7%) (Supplementary Figure S8), IL-6 (SMD = −1.94; 95% CI: −2.79, −1.10; *p* = 0.000; P_He_ < 0.001; I^2^ = 94.9%) (Supplementary Figure S9), IL-10 (SMD = −0.96; 95% CI: −1.29, −0.64; *p* = 0.000; P_He_ = 0.507; I^2^ = 0%), and TNF-α (SMD = −1.32; 95% CI: −1.96, −0.67; *p* = 0.000; P_He_ < 0.001; I^2^ = 89.7%) (Supplementary Figure S10).

### Effect of Hydroxyl Safflower Yellow A on Biomarkers of Oxidative Stress

The indicators of the effect of HSYA on oxidative stress in patients with DKD included SOD, MDA, and GSH-Px. A total of three articles reported that HSYA can produce a change in SOD levels (SMD = 0.76; 95% CI: 0.00, 1.52; *p* = 0.050; P_He_ < 0.001; I^2^ = 89.9%) (Supplementary Figure S11). Consistently, a total of four articles assessed whether HSYA can decrease MDA levels (SMD = −1.63; 95% CI: −2.69, −0.57; *p* = 0.003; P_He_ < 0.001; I^2^ = 95.0%) (Supplementary Figure S12). Only two articles evaluated the effects of HSYA on GSH-Px (SMD = 0.88; 95% CI: 0.57, 1.19; *p* = 0.000; P_He_ < 0.001; I^2^ = 92.7%), which indicated that HSYA increased GSH-Px levels. According to the results, we can conclude that HSYA therapy may have a significant effect on increasing SOD and GSH-Px levels and decreasing MDA levels in DKD. Publication bias was not conducted due to the limited number of articles included.

### Effect of Hydroxyl Safflower Yellow A on Renal Indicators

Compared with the control group, the pooled results of 13 trials showed that HSYA could significantly decrease BUN levels (SMD = −1.67; 95% CI: −2.25, −1.08; *p* = 0.000; P_He_ < 0.001; I^2^ = 94.6%) (Supplementary Figure S13). The pooled results of 24 groups assessing the UAER indicated a significant decrease (SMD = −1.58; 95% CI: −2.04, −1.12; *p* = 0.000; P_He_ < 0.001; I^2^ = 94.4%) (Supplementary Figure S14). Similarly, 15 groups reported that the HSYA group had a lower SCR (SMD = −0.77; 95% CI: −1.27, −0.26; *p* = 0.003; P_He_ < 0.001; I^2^ = 94.4%) (Supplementary Figure S15). However, there was significant heterogeneity in the results of all the aforementioned indicators.

### Publication Bias

We used Begg’s test and Egger’s test to detect publication bias. There were no publication biases for FBG by Begg’s test (*p* = 0.208) and Egger’s test (*p* = 0.206), for PBG by Begg’s test (*p* = 0.322) and Egger’s test (*p* = 0.227), for TNF-α by Begg’s test (*p* = 0.624) and Egger’s test (*p* = 0.248), and for SCR by Begg’s test (*p* = 0.216) and Egger’s test (*p* = 0.053). There were significant publication biases for hsCRP by Begg’s test (*p* = 0.012) and Egger’s test (*p* = 0.005), for IL-6 by Egger’s test (*p* = 0.009), for BUN by Begg’s test (*p* = 0.002) and Egger’s test (*p* = 0.000), and for the UAER by Begg’s test (*p* = 0.000) and Egger’s test (*p* = 0.003). We used the Duval and Tweedie nonparametric/trim and fill method in missing theoretical studies. However, the pooled SMDs of BUN, IL-6, hsCRP, and UAER were not significantly changed. We did not detect publication bias for IL-10, SOD, MDA, TG, TC, HbA1c, HOMA-IR, FINS, IL-10, and GSH-Px due to the limited number of studies.

### Subgroup Analysis and Sensitivity Analysis

We performed subgroup analysis by dosage and duration to explore the possible source of heterogeneity (see Supplementary Table S3 for details). We did not perform the subgroup analysis for the limited number of included studies in some indicators. According to the results of subgroup analysis, we found that when the dosage of HSYA was ≤ 100 mg or the duration was ≤ 4 weeks, the pooled results were more significant than when the dosage of HSYA was > 100 mg or the duration was > 4 weeks, which applies to other indicators as well, such as hsCRP, BUN, and UAER. For the underrepresentation in trials, whether the duration or dosage made a difference in IL-10, TNF-α, HOMA-IR, TC, TG, and MDA levels is unclear. It was suggested that the heterogeneity remained high for those indicators, including hsCRP, IL-6, TNF-α, FBG, BUN, UAER, and SCR. The heterogeneity was reduced when the dosage of HSYA was >100 mg, and the duration was >4 weeks for the UAER. When the duration was <4 weeks, heterogeneity for FBG, TG, and TC was reduced.

Additionally, we found that subgroup analysis failed to identify changes in FBG when the dosage of HSYA was >100 mg (SMD = −0.37; 95% CI: −1.32, 0.57; *p* = 0.437; I^2^ = 86.1%; P_He_ = 0.007) or when the duration was <4 weeks (SMD = −0.52; 95% CI: −1.17, 0.12; *p* = 0.112; I^2^ = 93.5%; P_He_ < 0.001). The effect of HSYA on UAER was not significantly different when the duration was 3 weeks (SMD = −0.70; 95% CI: −2.70, 1.29; *p* = 0.499; I^2^ = 98.0%; P_He_ < 0.001). The effect of HSYA on SCR was not significantly different when the duration was <4 weeks (SMD = −0.58; 95% CI: −1.36, 0.21; *p* = 0.148; I^2^ = 95.4%; P_He_ < 0.001).

In addition, we performed sensitivity analysis for all the included indicators. More details are shown in Supplementary Table S3. The results for sensitivity-pooled SMD were not significant for PBG when excluding the study reported by Guo (2020) and for SOD as well as FINS when excluding the study reported by Bao et al. (2017a).

### Quality Assessment

**FIGURE 2:**
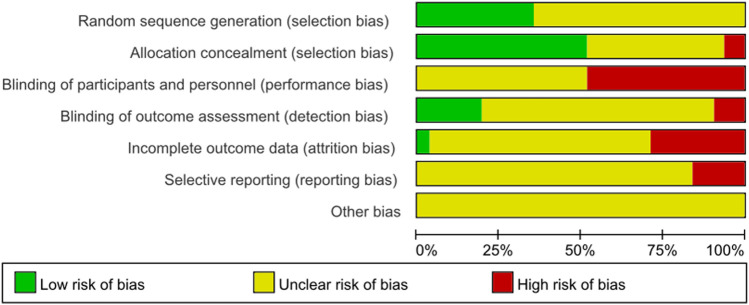
Methodological quality of the included studies (risk of bias).

**FIGURE 3:**
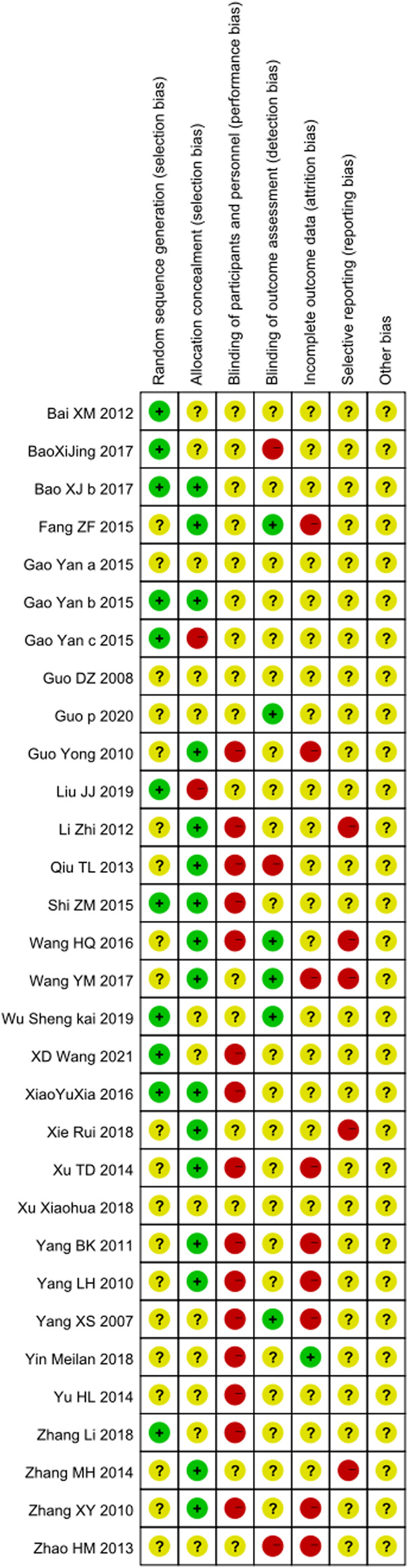
Methodological quality of the included studies (risk of bias).

We assessed all the included studies' methodological quality and bias by using the Cochrane Collaborations tool. The ROB summary is shown in Figure 2 and Figure 3. Overall, the included studies were of low quality.

## Discussion

DKD is characterized by a complex interaction of hemodynamic and metabolic factors, including overproduction of advanced glycation end-products (AGEs) and increased expression of proinflammatory cytokines such as interleukin-1 (IL-1), interleukin-6 (IL-6), and tumor necrosis factor-α (TNF-α) (Mottl et al., 2022; Navarro-González et al., 2011a). For the etiology of DKD at the cellular and molecular levels, oxidative stress and inflammatory reactions are currently considered critical pathophysiological mechanisms in the progression of DKD (Navarro-González et al., 2011a; Navarro-González et al., 2011b; Arora and Singh, 2014). Therefore, by targeting the inflammatory mechanisms and oxidative stress involved in DKD, more innovative anti-inflammatory and antioxidant therapeutic strategies have been gradually identified.

To optimize therapeutic strategies in DKD, increasing attention is given to Chinese herbal medicine (CHM) and its bioactive ingredients in DKD drug research. This, in combination with conventional treatments, can exert a pleiotropic action profile and may simultaneously obtain higher efficacy in treating DKD by intervening in the fundamental processes in the pathogenesis of DKD (Meresman et al., 2021). Hydroxyl safflower yellow A (HSYA), belonging to the monochalcone glycoside structure, is extracted from safflower (*Carthamus tinctorius* L.) and is a chief bioactive compound (Li et al., 2021a). Modern pharmacology and molecular biology studies have verified that HSYA can exert anti-inflammatory, antioxidant (He et al., 2021), and antiischemia reperfusion injury (Bai et al., 2020) effects and is widely used in the treatment of acute or chronic cardiovascular and cerebrovascular diseases (Xu et al., 2021; Geng et al., 2018). It has been shown to play a role in ameliorating cognitive impairment (Zhang et al., 2021a), rheumatic diseases (Tsiogkas et al., 2021), and osteoporosis (Wang et al., 2021b) and regulating glucose and lipid metabolism (Sun et al., 2021), providing new perspectives for exploring the development and application of HSYA in the health care industry (Sun et al., 2021).

This meta-analysis analyzed and summarized the levels of oxidative stress indicators and inflammatory mediators in patients with DKD. The pooled results indicated that the intravenous infusion of HSYA can significantly improve oxidative stress and the inflammatory response and could produce a better therapeutic effect than conventional Western medicine alone. The results in this meta-analysis also showed that the effect of HSYA can significantly reduce the level of SCR and UAER, which is consistent with the results reported by other meta-analyses (Jin et al., 2019; Wang et al., 2019b). Moreover, no adverse effects occurring in these clinical trials were reported by researchers. Therefore, the efficacy of HSYA on patients with DKD was significant and resulted in a better effect on antioxidant and anti-inflammatory indicators in combination with conventional Western medical treatment. Antioxidant and anti-inflammatory therapies of HSYA may contribute to relieving the degree of oxidative stress and inflammatory response and improving renal function, glucose metabolism, lipid metabolism, survival rate, and even the quality of life.

The potential relationship between oxidative stress and diabetes has generated considerable interest over the past decade. Oxidative stress is mainly due to the imbalance between oxidation and endogenous antioxidant capacity (Salim, 2014; Sureshbabu et al., 2015; Gyurászová et al., 2020), which can activate an inflammatory cascade, thus causing chronic local inflammatory stress in the kidney. The production of oxygen-free radicals increases in persistent hyperglycemia caused by autoxidation of glucose, which may further produce a series of disturbances in glucose and lipid metabolism and ultimately result in tissue and organ damage (Forman and Zhang, 2021; Sies, 2015). HSYA is regarded as a natural antioxidant with many beneficial effects in decreasing ROS and oxidative stress in cells. Several animal studies and cell studies demonstrated that HSYA could improve DKD by attenuating oxidative stress. In some animal studies, treatment with HSYA can significantly increase SOD and GSH-Px levels and protect kidney function in DKD in HFD- and STZ-induced rats while significantly decreasing malondialdehyde (MDA) in serum and renal tissues (Zhou et al., 2017; Lee et al., 2020; Zhao et al., 2021a; Zhao, 2021). In cell studies, HSYA was demonstrated to protect against renal podocyte damage (Xie and Zhou, 2021) and injury in glomerular mesangial cells (Xiao et al., 2016) induced by high glucose by inhibiting oxidative stress. Furthermore, HSYA can have a protective effect on H_2_O_2_-induced oxidative stress injury of endothelial cells (Cui et al., 2019) and can retard the calcification of vascular smooth muscle cells induced by hyperphosphatemia through the inhibition of oxidative stress (Han, 2020). In human trials, mounting evidence has demonstrated that HSYA can attenuate oxidative stress in some acute or chronic diseases (Du and Deng, 2019; Li and Zhang, 2020; Liu et al., 2020), such as coronary heart disease and cerebral infarction, which is similar to the results of our meta-analysis. Based on the experiments mentioned earlier, we speculate that HSYA can have a renoprotective role in DKD, and the underlying mechanisms of HSYA in DKD may be linked to its antioxidant properties.

Inflammatory cytokines, including TNF-α, IL-10, and IL-6, are considered a cardinal pathogenetic mechanism in the genesis and progression of DKD (Navarro-González et al., 2011a). Compared with healthy controls, patients with DKD can secrete a higher serum and urinary level of TNF-α, indicating that TNF-α can be a potential prognostic biomarker in postponing the progression of DKD (Moriwaki et al., 2003). The results reported indicate that genetic variations at the IL-10 promoter increase the risk of DKD, which may be a potential DKD genetic-susceptibility locus (Mtiraoui et al., 2009). Moreover, compared with diabetic patients without DKD, those with DKD can present with higher serum IL-6 levels, and certain polymorphisms in the IL-6 gene are carried in patients with type 2 diabetes mellitus (T2DM), which can contribute to the development of DKD (Feigerlová and Battaglia-Hsu, 2017). Thus, by targeting these inflammatory pathways in the context of DKD, the therapeutic strategy of HSYA is translated into the treatment of DKD. Many researchers have demonstrated that HSYA can decrease inflammatory cytokine levels, such as TNF-α, nuclear factor-κB (NF-κB), IL-6, IL-β, and IL-10, in animal models of STZ-induced DKD (Lee et al., 2020; Zhao, 2021; Zhao et al., 2021b; Xing, 2021). Consistent with the results mentioned earlier, our meta-analysis also showed that HSYA could exert an anti-inflammatory effect in patients with DKD. In addition, HSYA can alleviate hsCRP serum levels. Therefore, we may conclude that HSYA can have a potential effect of lowering some inflammatory markers among patients with DKD. Nevertheless, for the limited studies included, the result in our meta-analysis showed that HSYA may contribute to lower IL-10, which is contrary to the anti-inflammatory properties of HSYA. Moreover, it is still necessary to conduct additional prospective studies to explore different doses of HSYA and different durations of supplementation and their effects on biomarkers of inflammation. Dyslipidemia and hyperglycemia together lead to a microinflammatory state and the production of reactive oxygen species (ROS), which can thus promote inflammation, oxidative stress, lipid peroxidation, and vesicular transport dysfunction (Martínez-García et al., 2015; Meex et al., 2019), further contributing to cellular damage. Thus, the regulation of lipid and glucose metabolism is critical for alleviating DKD. A series of studies have shown that HSYA can regulate glucose and lipid metabolism by inhibiting the apoptosis of pancreatic β-cells, improving insulin resistance, and regulating glycolipid metabolism, which is related to its antioxidant and anti-inflammatory activities *in vitro* (Liao et al., 2019). In our meta-analysis, the results showed that HSYA can significantly reduce FBG, PBG, FINS, HOMA-IR, TG, TC, and HbA1c levels, contributing to the promotion of the use of HSYA in clinical practice for diabetes.

Multiple signaling pathways may be involved in the molecular mechanisms of antioxidant and anti-inflammatory HSYA properties in the treatment of DKD. Molecular targets and signaling pathways related to protecting the kidney from injury led by oxidative stress and inflammatory reactions caused by hyperglycemia might include the inhibition of protein kinase C-/rat sarcoma-rapidly accelerated fibrosarcoma-MEK-extracellular signal-regulated kinase (PKC/Ras-Raf-MEK-ERK) signaling pathway (Zhao, 2021), the inhibition of the c-Jun NH(2)-terminal kinase (JNK) signaling pathway activation (Xie and Zhou, 2021), the inhibition of vascular endothelial growth factor (VEGF) protein (Xie and Zhou, 2021), the inhibition of cleaved caspase-12, cleaved-PARP, CHOP, p-eIF2α, GRP78, and miR-302a-3p (Zhang et al., 2021b), the inhibition of miR-516a-5p (Li et al., 2021b), and the activation of sirtuin1-nuclear factor-erythroid 2-related factor 2 (SIRT1-Nrf2) (Zhang, 2020). To have a good logic flow, a figure is given to indicate how HSYA benefits on DKD (Supplementary Image S1). Overall, the antioxidant and anti-inflammatory properties of HSYA could intervene in the progression of DKD through multiple signaling pathways.

Several limitations should be considered in this systematic review and meta-analysis. Significant heterogeneity was found in most pooled results, and subgroup analysis by dosage and duration hardly reduced the heterogeneity and the ultimate pooled results on most targeted outcomes. However, the duration can affect the stability of the results on SCR, UAER, and FBG outcomes, thus deserving further study. The methodological quality of the included studies was low, which may partly reduce the reliability of the pooled studies. In addition, it is unclear whether different doses of HSYA influence the therapeutic effect on IL-6, IL-10, TNF-α, HOMA-IR, and MDA levels. Furthermore, a firm conclusion cannot be drawn from the limited number of articles, and small populations included some biomarkers of inflammation and oxidative stress, such as SOD, MDA, IL-10, and GSH-Px. The included populations were mainly Chinese, which may also hinder the promotion of this treatment for other races. Based on our findings, it is necessary to explore the effect of HSYA and the association between dose and intervention duration and adverse effects by following the strict methodology of RCTs and by including more populations. Last but not least, for the treatment of DKD, it is imperative to pay attention to long-term patient management; intravenous administration can significantly reduce patient compliance, and no clinical studies have been conducted on the oral effect of HSYA in patients with DKD.

## Conclusion

It is prudent to develop and apply HSYA to treat and manage DKD. By improving biomarkers of oxidative stress and the inflammatory response, HSYA can efficiently attenuate the overactivation of ROS, inhibit the production of proinflammatory cytokines, regulate blood glucose and lipid levels, and improve renal function indices. The mechanism of action of HSYA in patients with DKD may be linked to its beneficial antioxidant and anti-inflammatory effects. Exploration of the cellular and molecular basis of signaling pathways will promote the application of HSYA for patients with DKD and contribute to developing more and better treatments for DKD. However, a multicenter, multiethnic, high-quality randomized clinical trial is still needed in the future to further demonstrate the effect of HSYA on oxidative stress and inflammatory mediators in patients with DKD. It is also imperative to conduct a study on the oral administration of HSYA.

## Data Availability

The original contributions presented in the study are included in the article/Supplementary Material; further inquiries can be directed to the corresponding author.

## Glossary

RAAS: renin angiotensin aldosterone system
PKC: protein kinase C: protein kinase C
TGF-β: transforming growth factor-β
TNF-α: tumor necrosis factor-α
ESRD: end-stage renal disease
DKD: diabetic kidney disease
eGFR: estimated glomerular filtration rate
ROS: reactive oxygen species: reactive oxygen species
HSYA: hydroxyl safflower yellow A
UAER: urinary albumin excretion rate
FBG: fasting blood glucose
SCR: serum creatinine
BUN: blood urea nitrogen
SRs/MAs: systematic review and meta-analysis
ROS: reactive oxygen species: reactive oxygen species
WM: Western medicine
SOD: superoxide dismutase
GSH-Px: glutathione peroxidase
MDA: malondialdehyde
PBG: postprandial blood glucose
HbA1c: hemoglobin A1c
RCTs: randomized controlled trials
FINS: fasting insulin
HOMA-IR: homeostasis model assessment insulin resistance
Cysc: serum cystatin C
Hcy: homocysteine
PKC: protein kinase C: protein kinase C
IL-1β: interleukin-1β
IL-6: interleukin-6
NF-κB: nuclear factor-κB
IL-10: interleukin-10
hsCRP: hypersensitive c reactive protein
total cholesterol: TC
triglycerides: TGs
