# Supplementary material for: Antioxidant and Anti-Inflammatory Properties of Hydroxyl Safflower Yellow a in Diabetic Nephropathy: A Meta-Analysis of Randomized Controlled Trials
Source: Front Pharmacol. 2022 Aug 11;13:929169. doi: 10.3389/fphar.2022.929169 (PMC9404325; doi:10.3389/fphar.2022.929169)
Supplement: Supplementary file 1 [file DataSheet7.pdf]

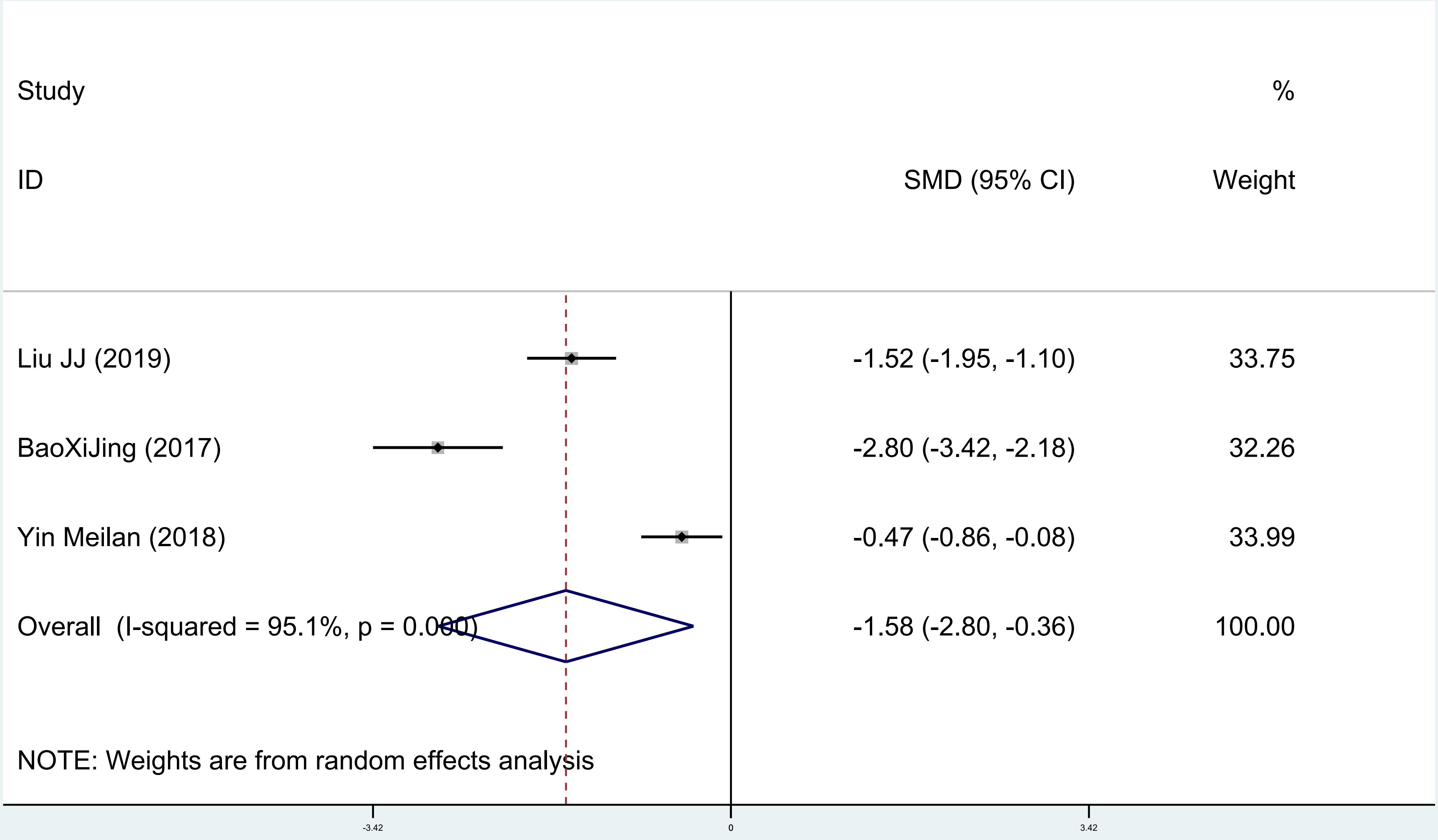

Study
%
ID
SMD (95% CI)
Weight
Liu JJ (2019)
-1.52 (-1.95, -1.10)
33.75
BaoXiJing (2017)
-2.80 (-3.42, -2.18)
32.26
Yin Meilan (2018)
-0.47 (-0.86, -0.08)
33.99
Overall (I-squared = 95.1%, p = 0.000)
-1.58 (-2.80, -0.36)
100.00
NOTE: Weights are from random effects analysis
-3.42
0
3.42
