# Supplementary figures and images for: Antioxidant and Anti-Inflammatory Properties of Hydroxyl Safflower Yellow a in Diabetic Nephropathy: A Meta-Analysis of Randomized Controlled Trials
Source: Front Pharmacol. 2022 Aug 11;13:929169. doi: 10.3389/fphar.2022.929169 (PMC9404325; doi:10.3389/fphar.2022.929169)

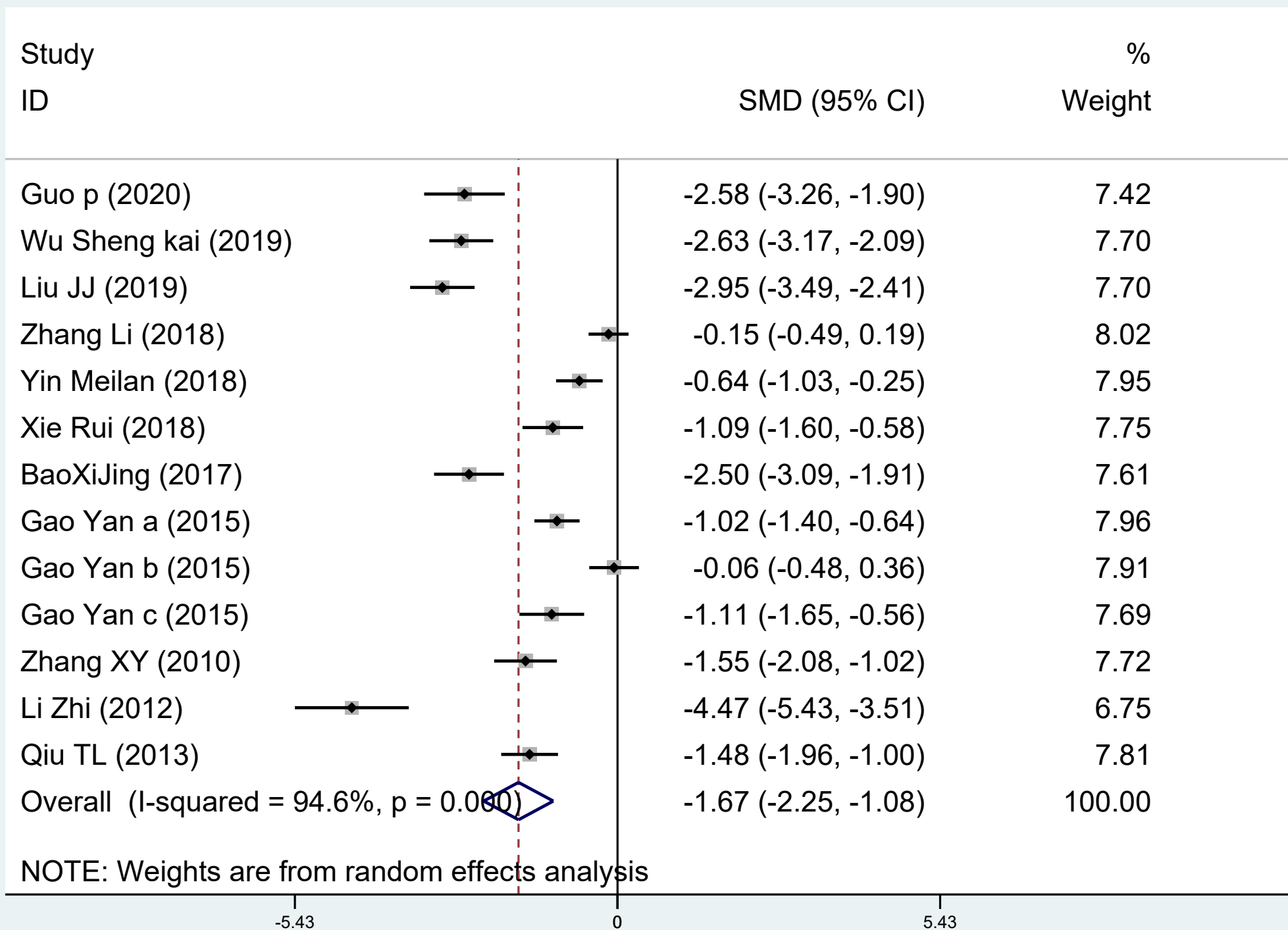

Supplement: Supplementary file 2 [file DataSheet13.pdf]

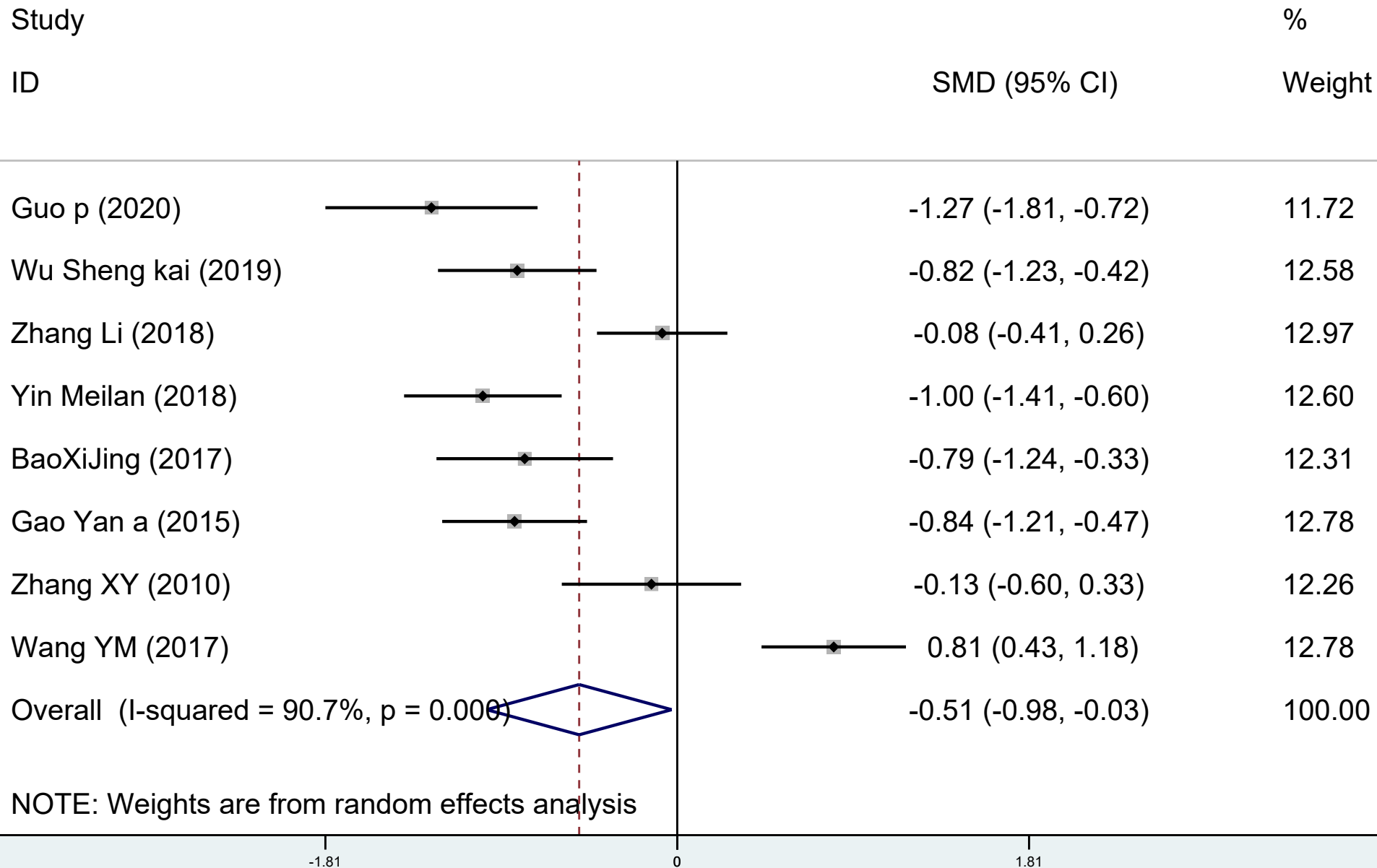

Supplement: Supplementary file 3 [file DataSheet2.pdf]

Study %

ID SMD (95% CI) Weight

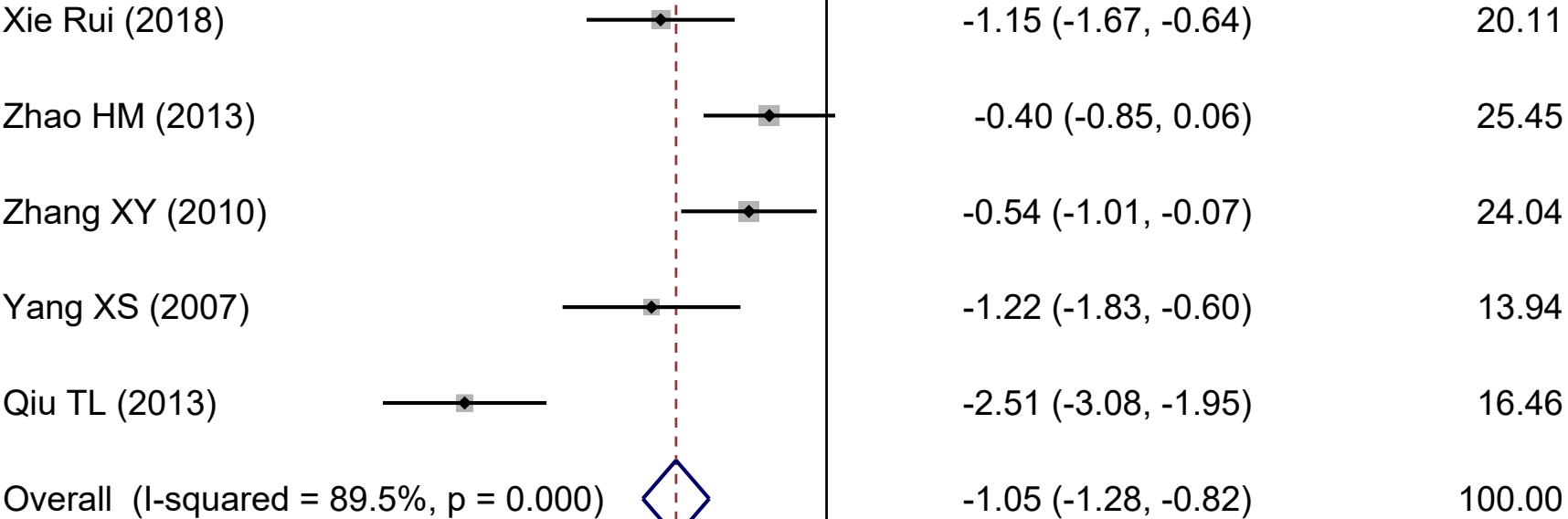

-3.08

0

3.08

Supplement: Supplementary file 5 [file DataSheet6.pdf]

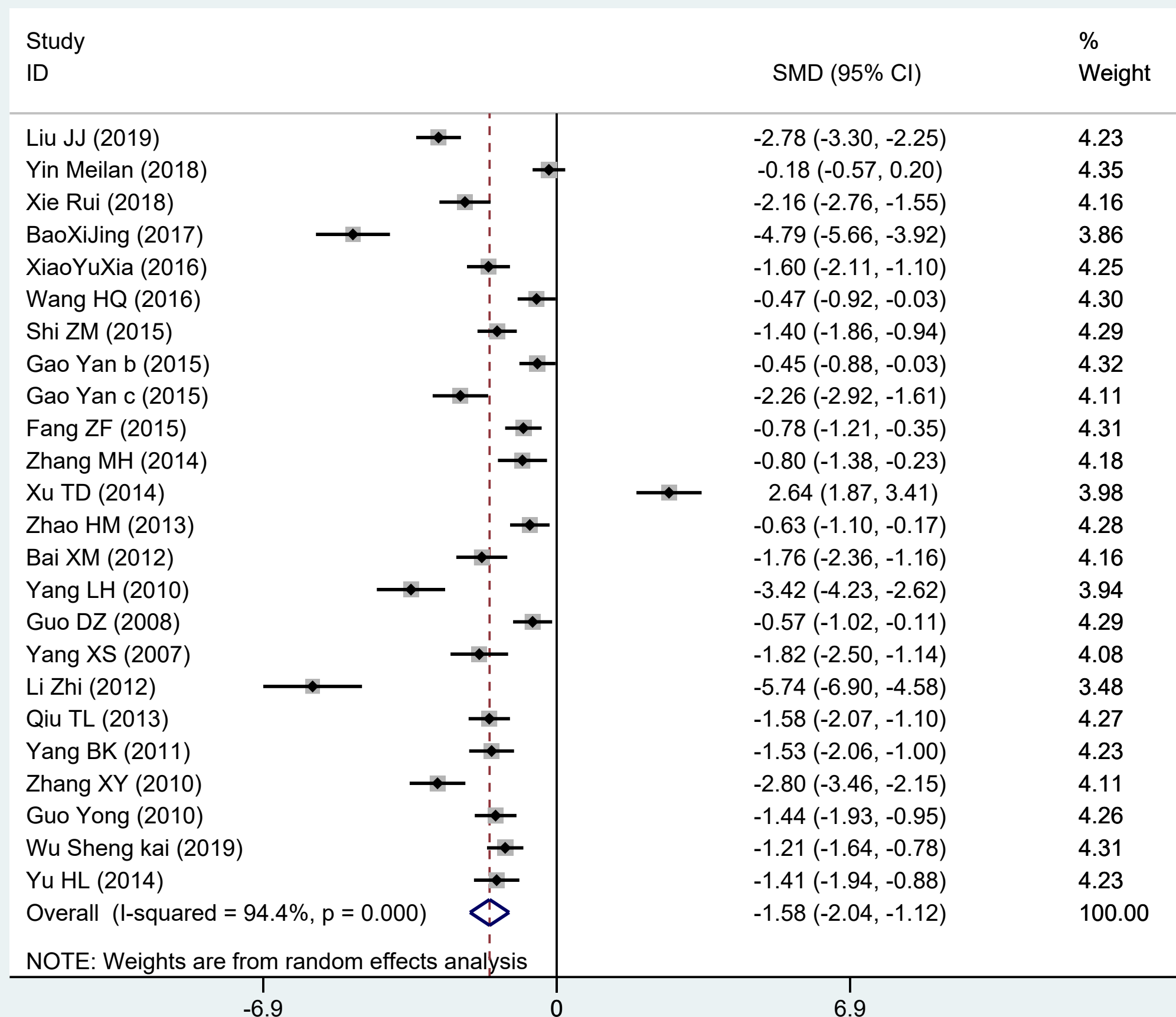

Supplement: Supplementary file 6 [file DataSheet14.pdf]

Study

ID

SMD (95% CI)

%  
Weight

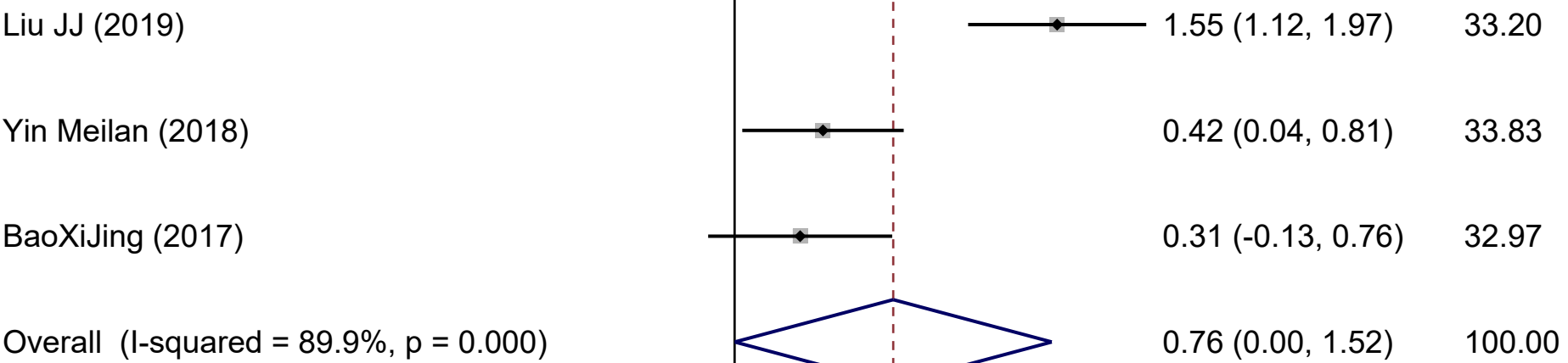

NOTE: Weights are from random effects analysis

Supplement: Supplementary file 8 [file DataSheet11.pdf]

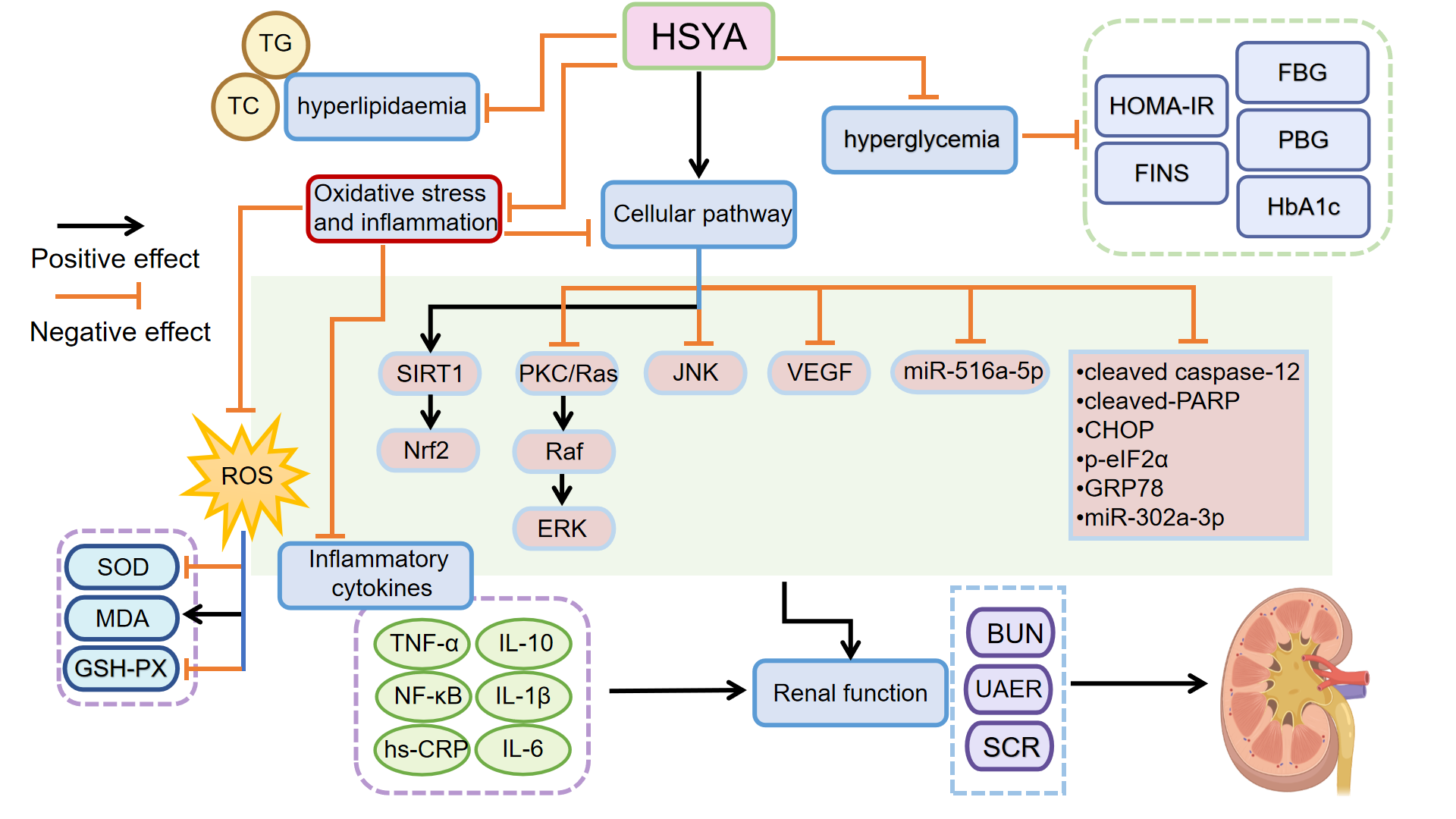

Supplement: Supplementary file 9 [file Image1.tif]

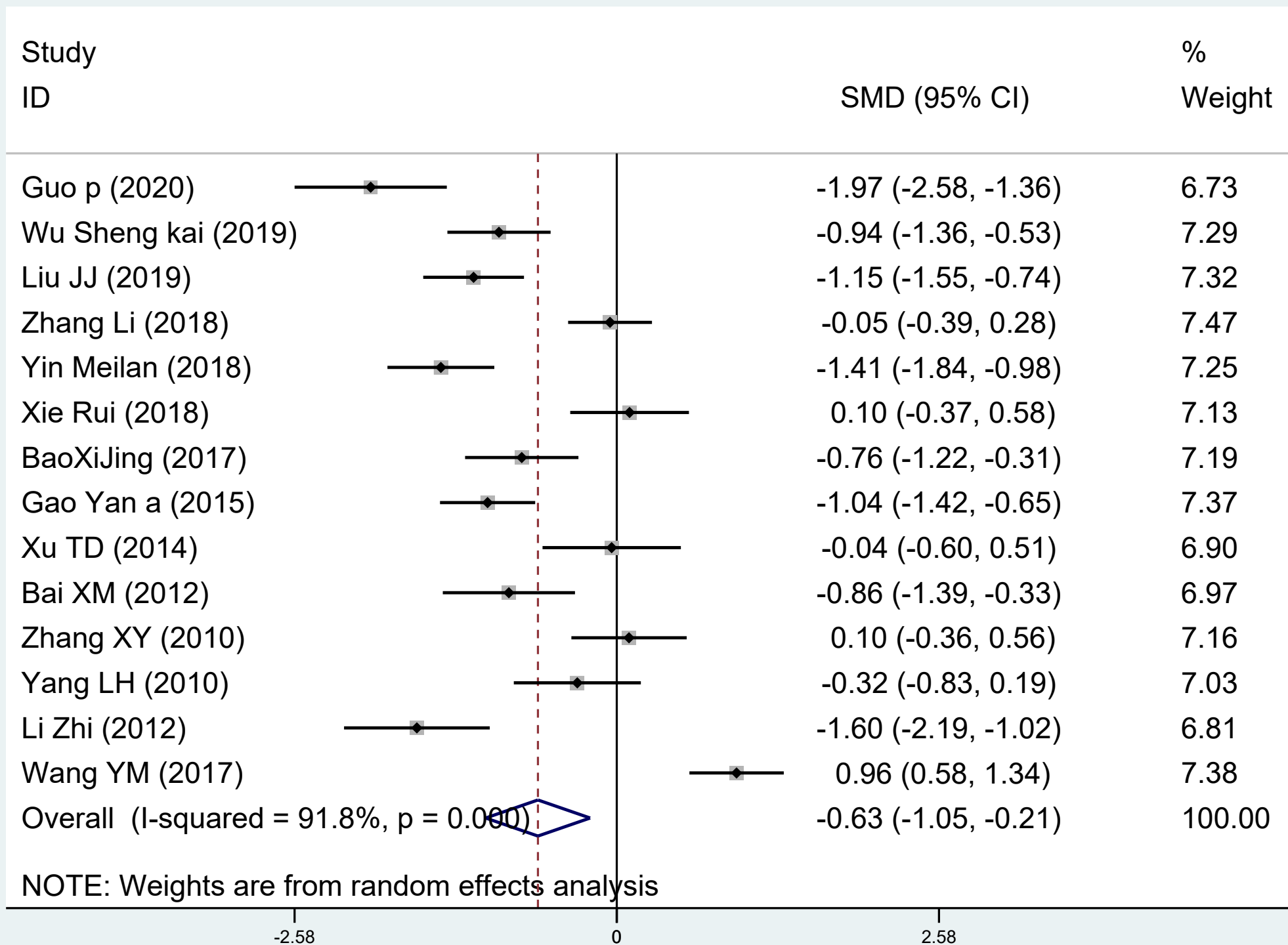

Supplement: Supplementary file 11 [file DataSheet1.pdf]

Study

%

ID

SMD (95% CI)

Weight

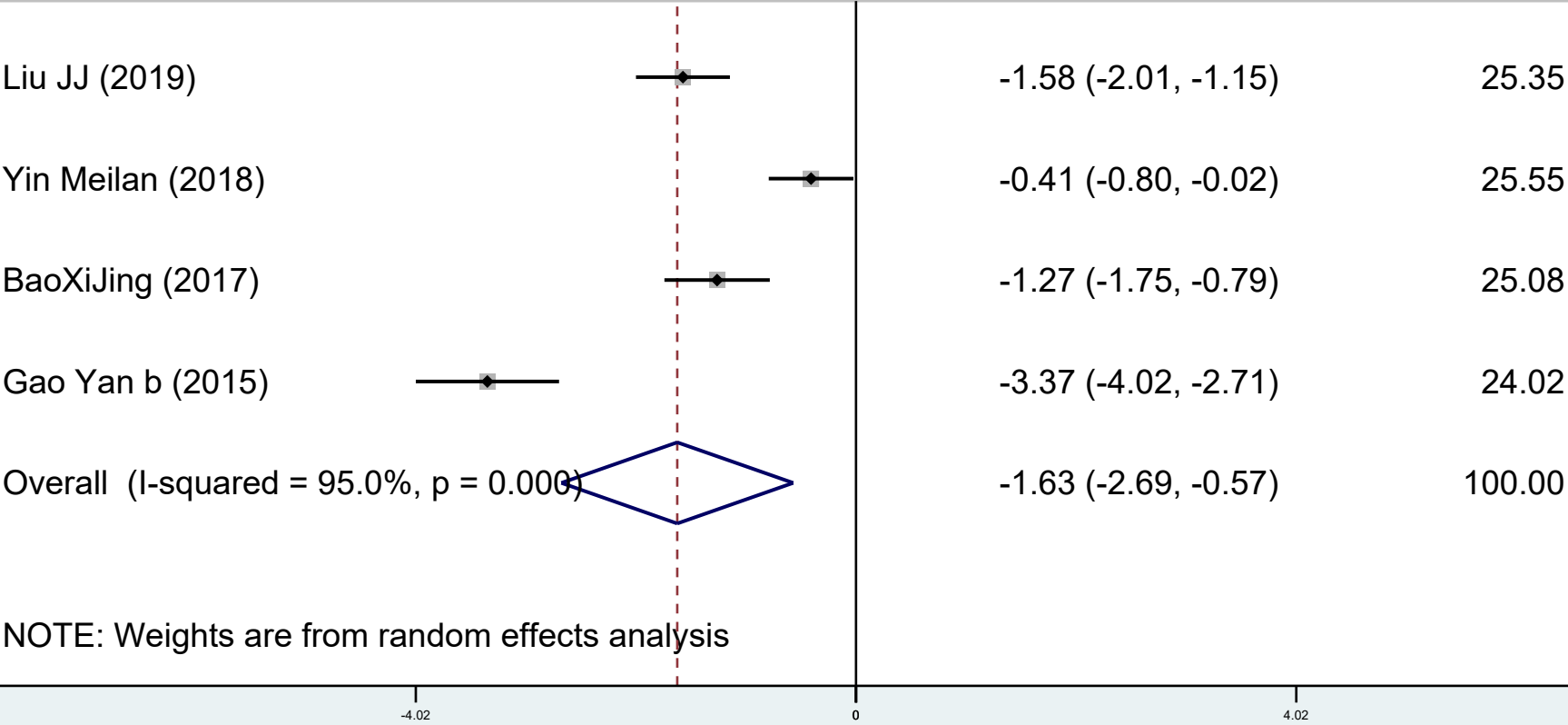

Supplement: Supplementary file 14 [file DataSheet12.pdf]

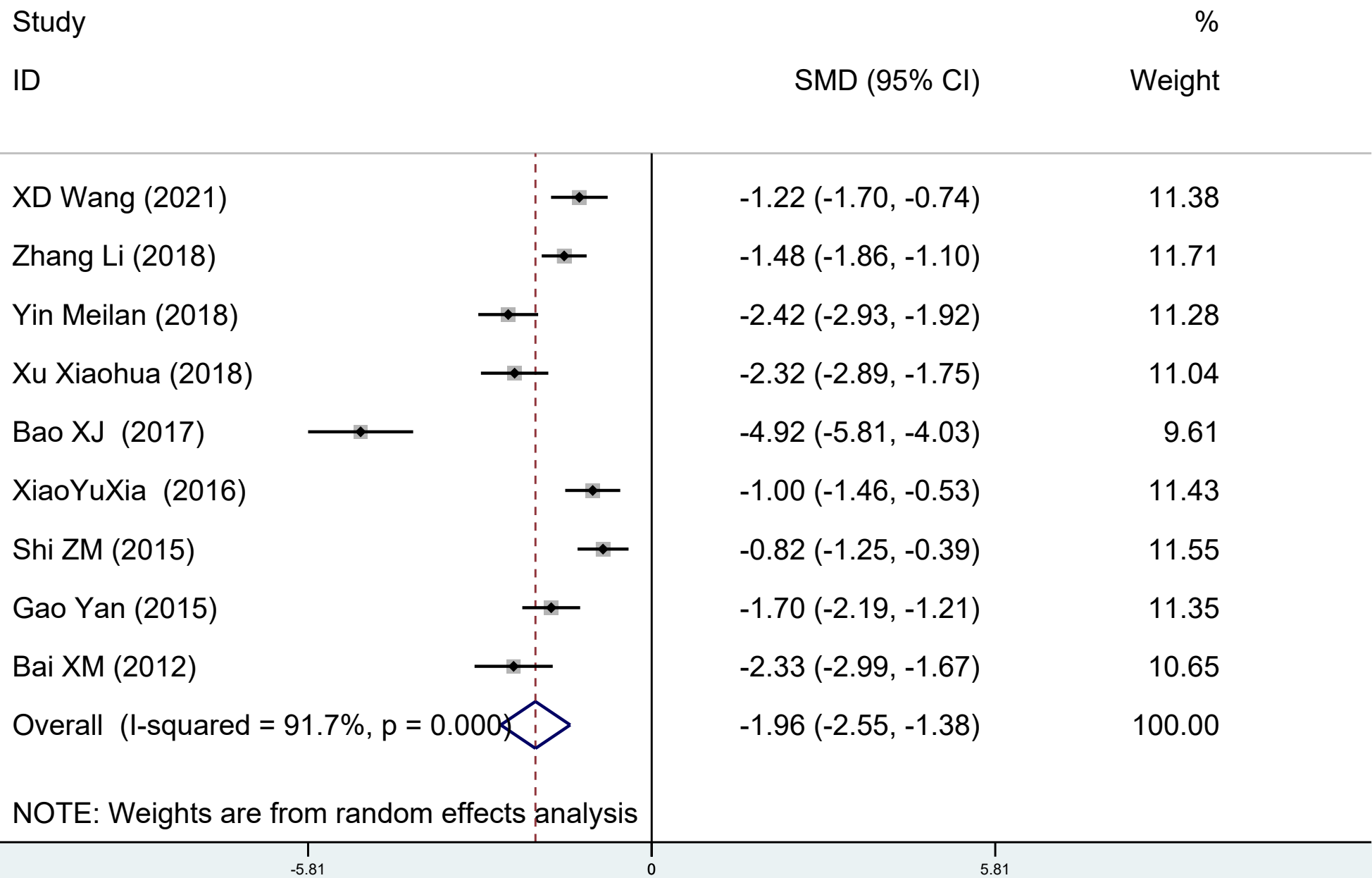

Supplement: Supplementary file 15 [file DataSheet8.pdf]

Study

%

ID

SMD (95% CI)

Weight

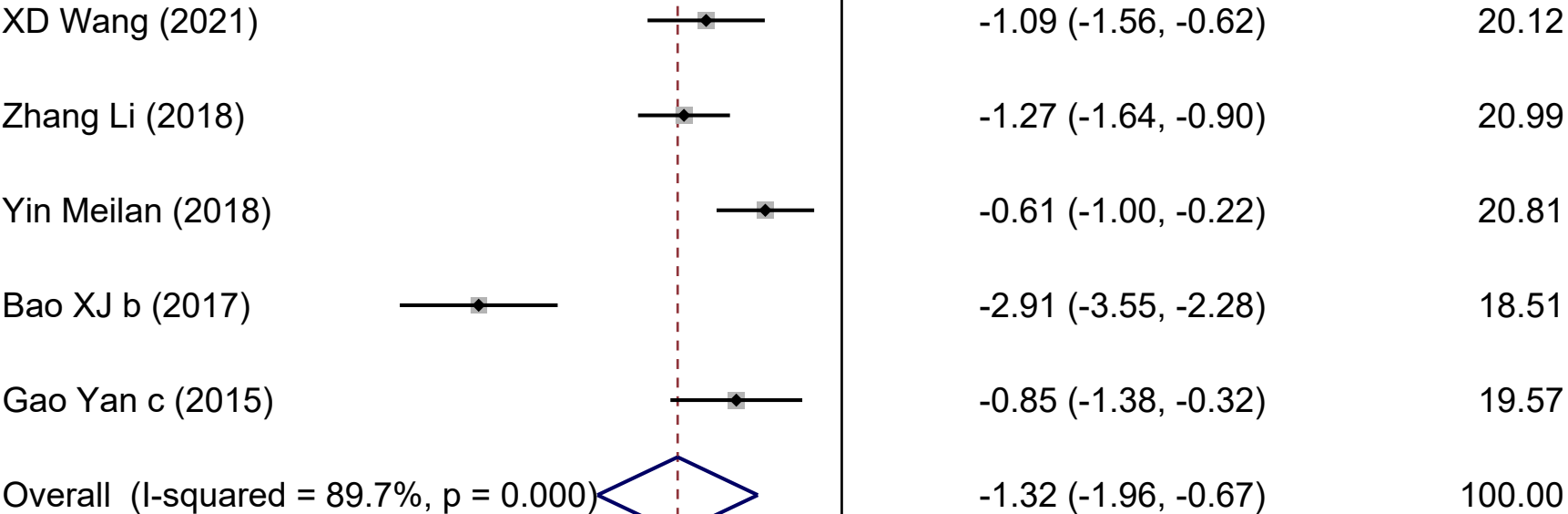

NOTE: Weights are from random effects analysis

Supplement: Supplementary file 16 [file DataSheet10.pdf]

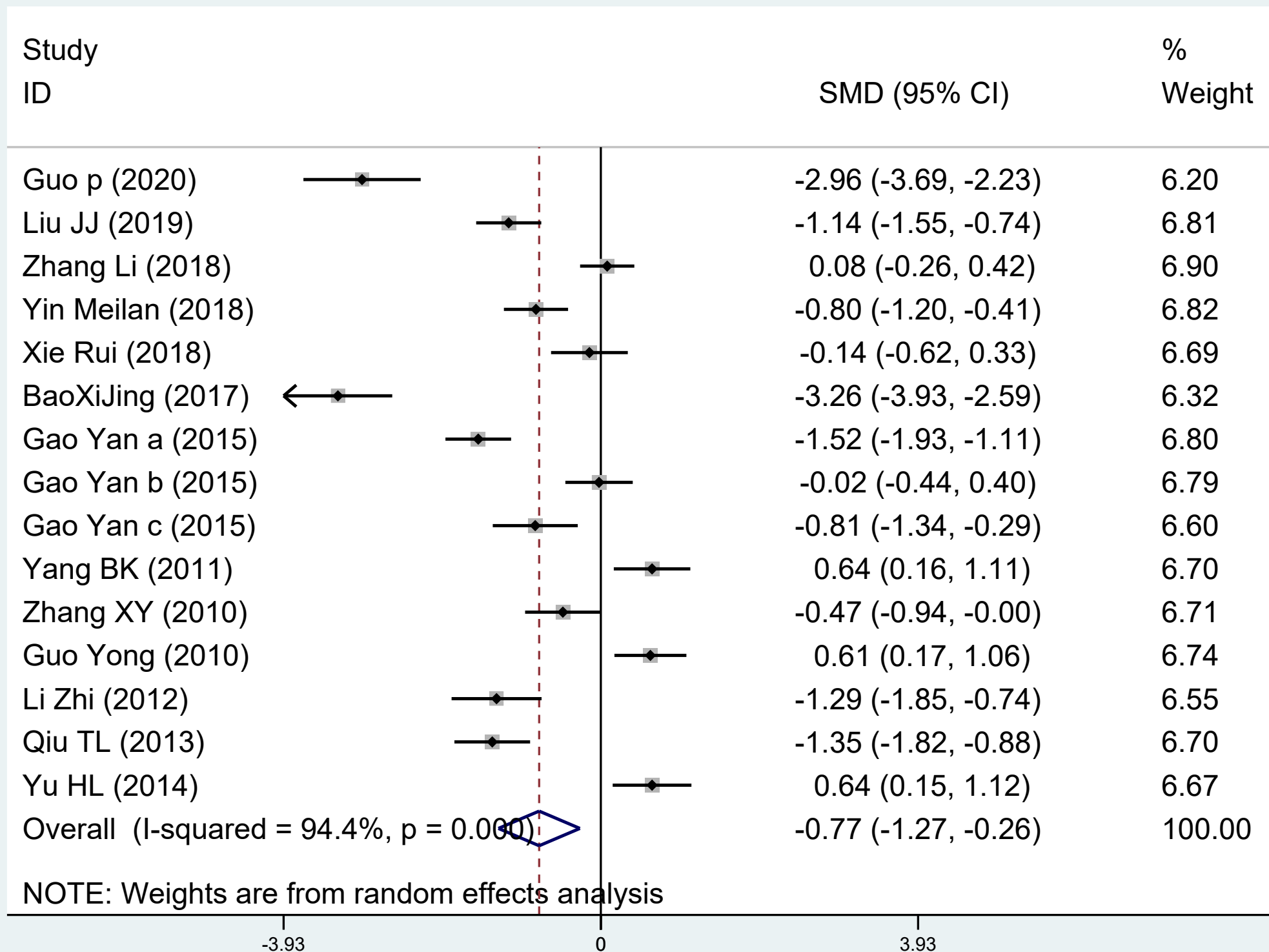

Supplement: Supplementary file 17 [file DataSheet15.pdf]
