# Supplementary material for: Antioxidant and Anti-Inflammatory Properties of Hydroxyl Safflower Yellow a in Diabetic Nephropathy: A Meta-Analysis of Randomized Controlled Trials
Source: Front Pharmacol. 2022 Aug 11;13:929169. doi: 10.3389/fphar.2022.929169 (PMC9404325; doi:10.3389/fphar.2022.929169)
Supplement: Supplementary file 4 [file DataSheet4.pdf]

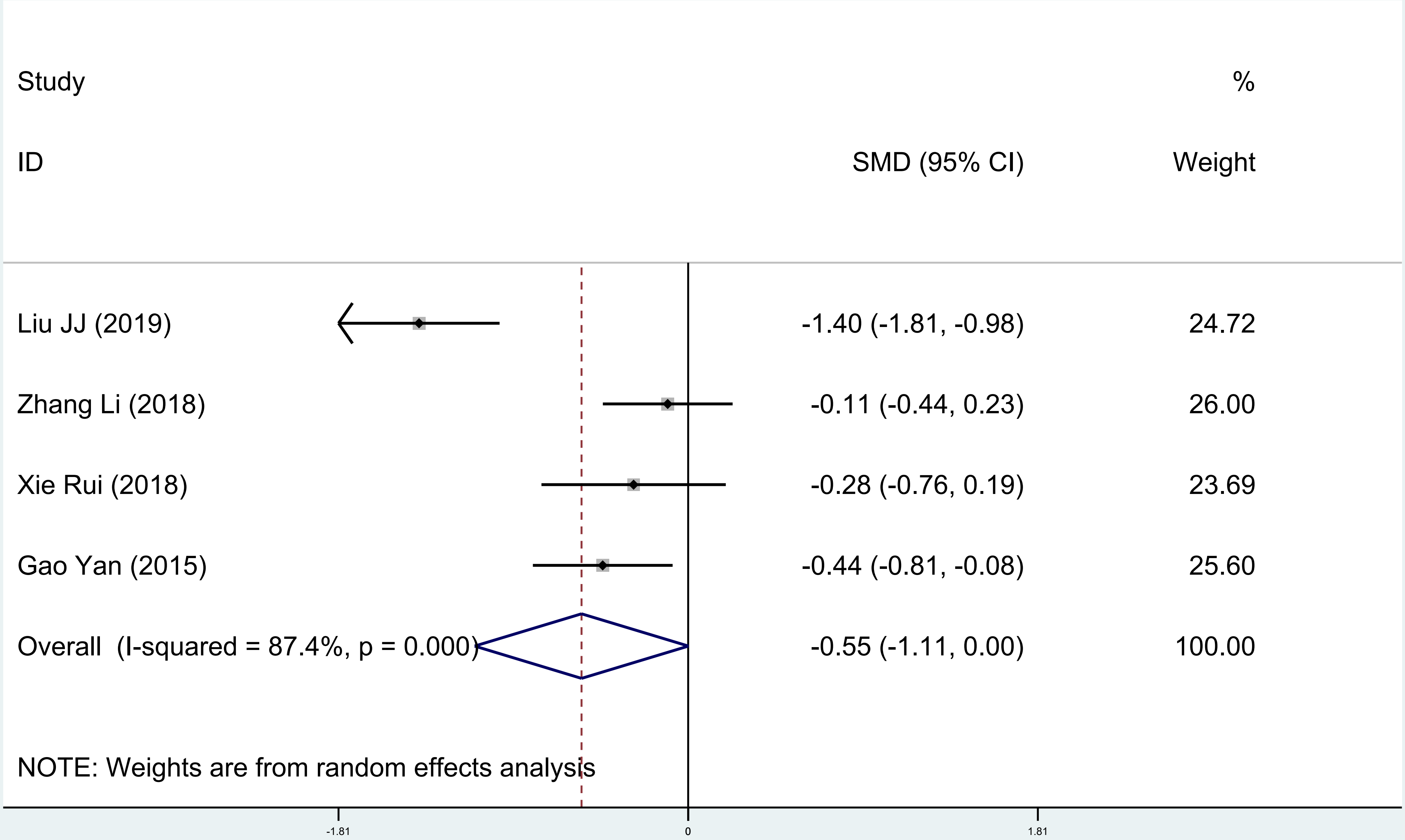

Study
ID
%
SMD (95% CI)
Weight
Liu JJ (2019)
-1.40 (-1.81, -0.98)
24.72
Zhang Li (2018)
-0.11 (-0.44, 0.23)
26.00
Xie Rui (2018)
-0.28 (-0.76, 0.19)
23.69
Gao Yan (2015)
-0.44 (-0.81, -0.08)
25.60
Overall (I-squared = 87.4%, p = 0.000)
-0.55 (-1.11, 0.00)
100.00
NOTE: Weights are from random effects analysis
-1.81
0
1.81
