# Supplementary material for: Antioxidant and Anti-Inflammatory Properties of Hydroxyl Safflower Yellow a in Diabetic Nephropathy: A Meta-Analysis of Randomized Controlled Trials
Source: Front Pharmacol. 2022 Aug 11;13:929169. doi: 10.3389/fphar.2022.929169 (PMC9404325; doi:10.3389/fphar.2022.929169)
Supplement: Supplementary file 7 [file DataSheet9.pdf]

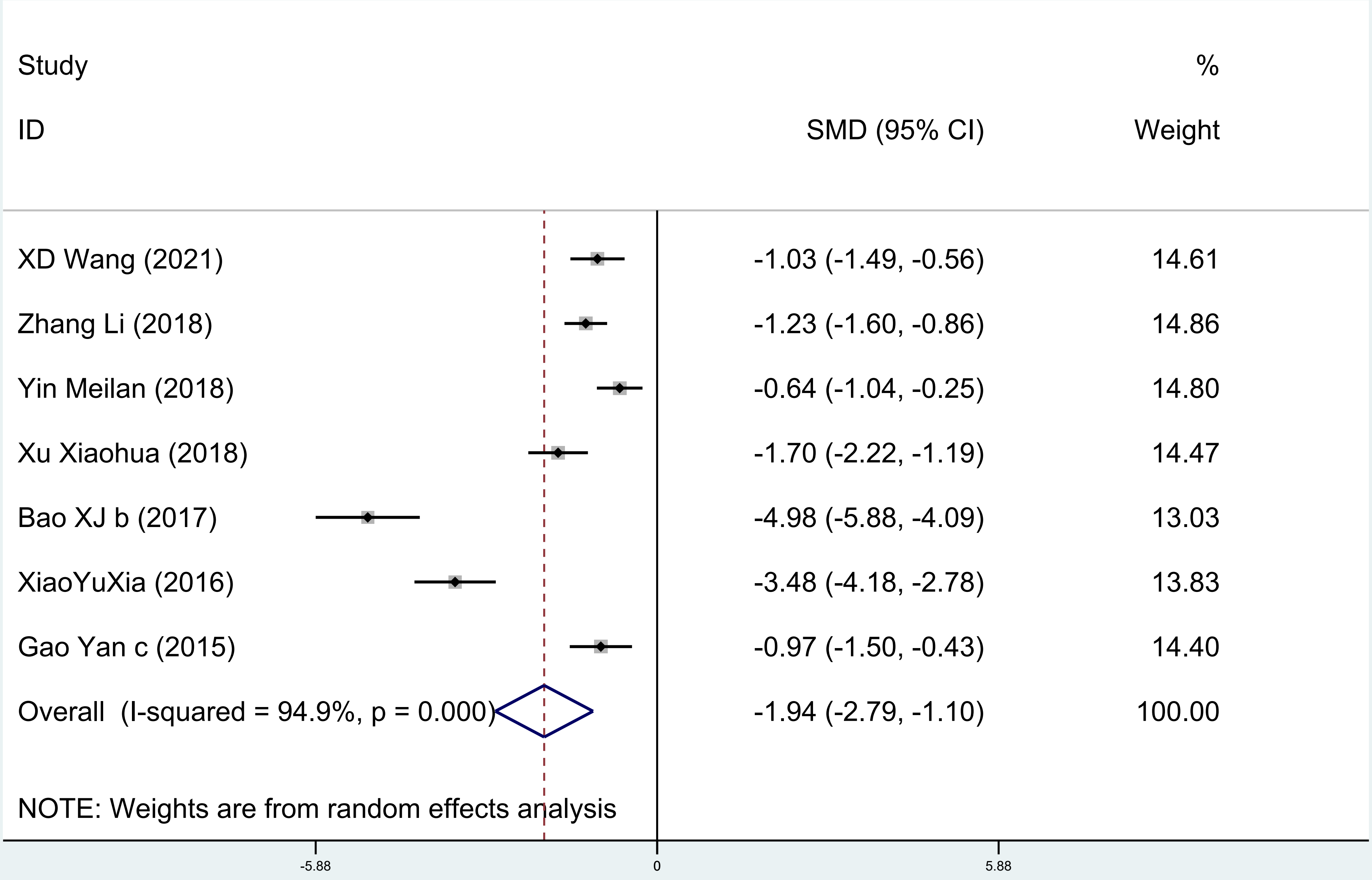
Study
ID
%
Weight
SMD (95% CI)
XD Wang (2021)
-1.03 (-1.49, -0.56)
14.61
Zhang Li (2018)
-1.23 (-1.60, -0.86)
14.86
Yin Meilan (2018)
-0.64 (-1.04, -0.25)
14.80
Xu Xiaohua (2018)
-1.70 (-2.22, -1.19)
14.47
Bao XJ b (2017)
-4.98 (-5.88, -4.09)
13.03
XiaoYuXia (2016)
-3.48 (-4.18, -2.78)
13.83
Gao Yan c (2015)
-0.97 (-1.50, -0.43)
14.40
Overall (I-squared = 94.9%, p = 0.000)
-1.94 (-2.79, -1.10)
100.00
NOTE: Weights are from random effects analysis
-5.88
0
5.88
