# Supplementary material for: Antioxidant and Anti-Inflammatory Properties of Hydroxyl Safflower Yellow a in Diabetic Nephropathy: A Meta-Analysis of Randomized Controlled Trials
Source: Front Pharmacol. 2022 Aug 11;13:929169. doi: 10.3389/fphar.2022.929169 (PMC9404325; doi:10.3389/fphar.2022.929169)
Supplement: Supplementary file 10 [file DataSheet3.pdf]

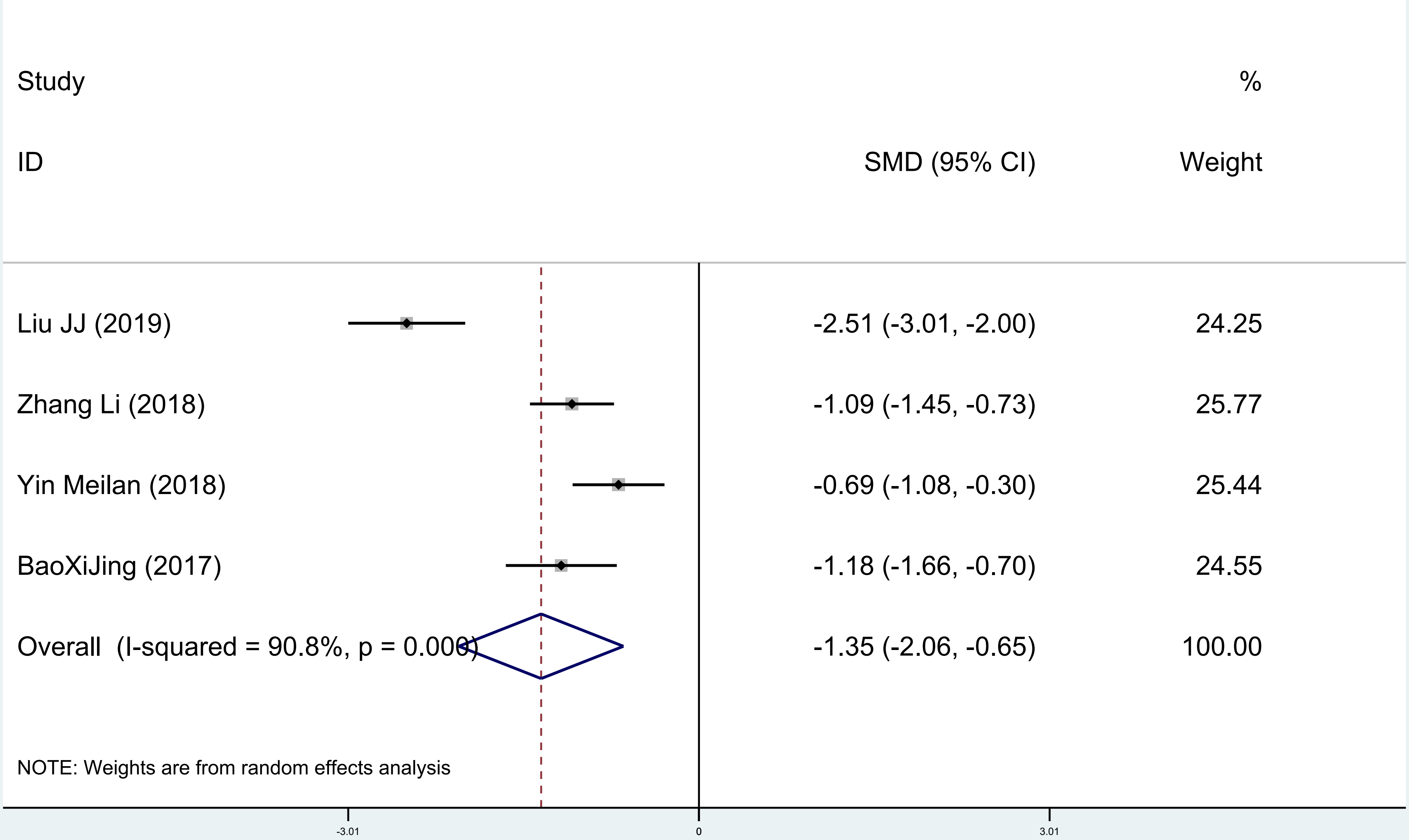

Study
ID
%
SMD (95% CI)
Weight
Liu JJ (2019)
-2.51 (-3.01, -2.00)
24.25
Zhang Li (2018)
-1.09 (-1.45, -0.73)
25.77
Yin Meilan (2018)
-0.69 (-1.08, -0.30)
25.44
BaoXiJing (2017)
-1.18 (-1.66, -0.70)
24.55
Overall (I-squared = 90.8%, p = 0.000)
-1.35 (-2.06, -0.65)
100.00
NOTE: Weights are from random effects analysis
-3.01
0
3.01
