# Supplementary material for: Antioxidant and Anti-Inflammatory Properties of Hydroxyl Safflower Yellow a in Diabetic Nephropathy: A Meta-Analysis of Randomized Controlled Trials
Source: Front Pharmacol. 2022 Aug 11;13:929169. doi: 10.3389/fphar.2022.929169 (PMC9404325; doi:10.3389/fphar.2022.929169)
Supplement: Supplementary file 12 [file DataSheet5.pdf]

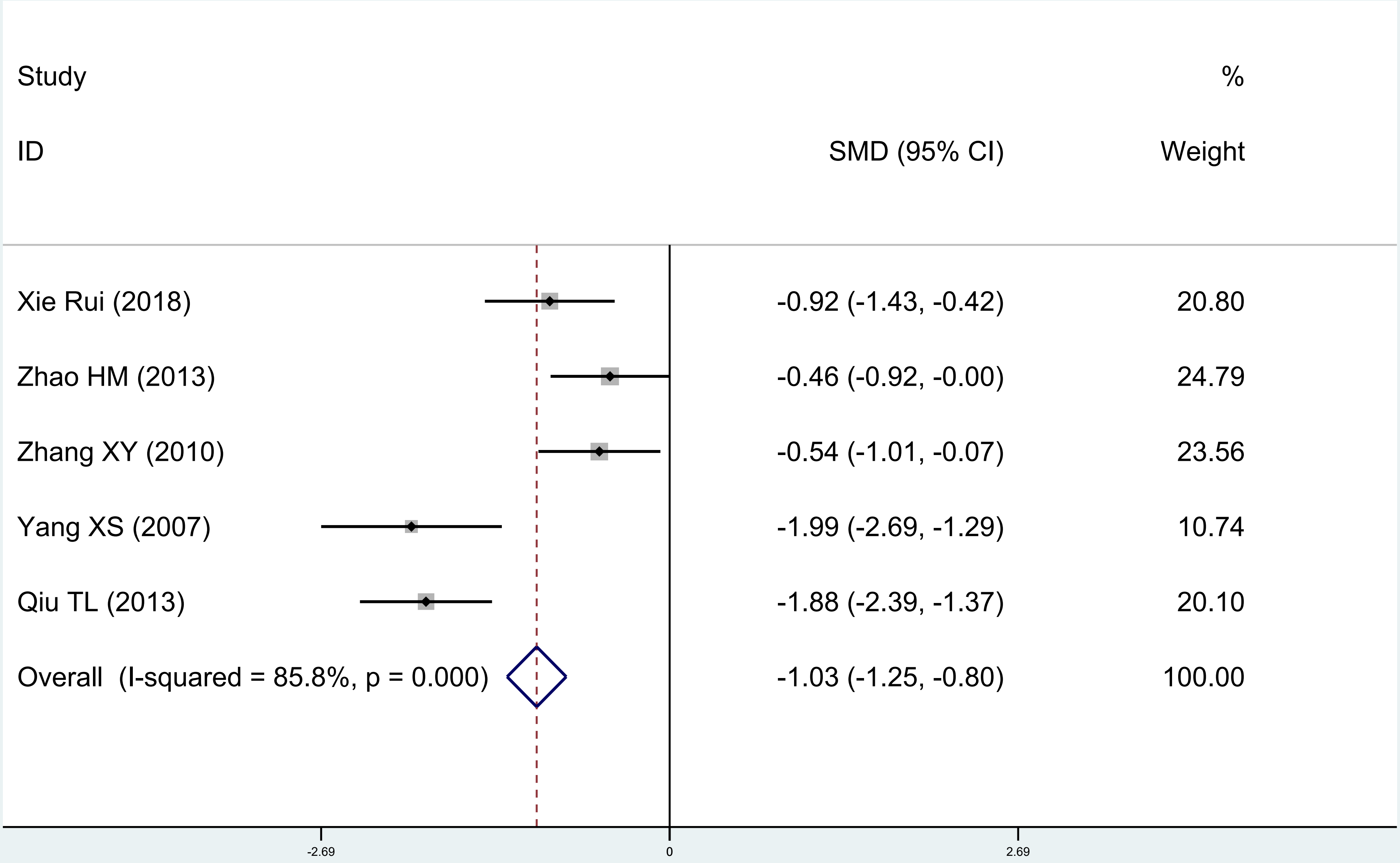
Study
ID
%
SMD (95% CI)
Weight
Xie Rui (2018)
-0.92 (-1.43, -0.42)
20.80
Zhao HM (2013)
-0.46 (-0.92, -0.00)
24.79
Zhang XY (2010)
-0.54 (-1.01, -0.07)
23.56
Yang XS (2007)
-1.99 (-2.69, -1.29)
10.74
Qiu TL (2013)
-1.88 (-2.39, -1.37)
20.10
Overall (I-squared = 85.8%, p = 0.000)
-1.03 (-1.25, -0.80)
100.00
-2.69
0
2.69
