# Supplementary material for: Antioxidant and Anti-Inflammatory Properties of Hydroxyl Safflower Yellow a in Diabetic Nephropathy: A Meta-Analysis of Randomized Controlled Trials
Source: Front Pharmacol. 2022 Aug 11;13:929169. doi: 10.3389/fphar.2022.929169 (PMC9404325; doi:10.3389/fphar.2022.929169)
Supplement: Supplementary file 13 [file Table3.docx]

| **Indicators** | **Lower pooled SMD** | **Excluding study** | **Higher pooled SMD** | **Excluding study** |
| --- | --- | --- | --- | --- |
| hsCRP | -2.11(-2.73,-1.40) | Shi ZM,2015 | -1.84(-2.0,-1.21) | BaoXiJing,2017 |
| TNF-α | -1.50(-2.25,-0.76) | Gao Yan b,2015 | -0.96(-1.27,-0.65) | Bao XJ b, 2017 |
| IL-6 | -2.18(-3.14,-1.21) | Yin Meilan,2018 | -1.47(-2.10,-0.64) | Bao XJ b, 2017 |
| FBG | -0.76(-1.10,-0.41) | Wang YM,2017 | -0.54(-0.96,-0.12) | Guo p, 2020 |
| PBG | -0.69(-1.02,-0.36) | Wang YM,2017 | -0.40(-0.90,0.09) | Guo p, 2020 |
| SCR | -0.86(-1.38,-0.35) | Yang BK,2011 | -0.59(-1.05,-0.13) | BaoXiJing,2017 |
| BUN | -1.80(-2.39,-1.21) | Zhang Li,2018 | -1.46(-2.01,-0.91) | Li Zhi, 2012 |
| UAER | -1.74(-2.15,-1.33) | Xu TD, 2014 | -1.42(-1.86,-0.99) | Li Zhi, 2012 |
| HbA1c | -0.71(-1.38,-0.04) | Zhang Li,2018 | -0.27(-0.49,-0.05) | Liu JJ,2019 |
| FINS | -2.14(-3.39,-0.89) | Yin Meilan,2018 | -0.99(-2.03,0.04) | BaoXiJing,2017 |
| HOMA-IR | -1.58(-2.43,-0.73) | Yin Meilan,2018 | -0.97(-1.27,-0.68) | Liu JJ,2019 |
| MDA | -2.05(-3.15,-0.95) | Yin Meilan,2018 | -1.08(-1.81,-0.35) | Gao Yan b,2015 |
| SOD | 0.38(0.09,0.67) | Liu JJ,2019 | 0.98(-0.12,2.08) | BaoXiJing,2017 |
| TG | -1.35(-2.17,-0.53) | Zhao HM,2013 | -0.80(-1.20,-0.39) | Qiu TL,2013 |
| TC | -1.31(-2.01,-0.61) | Zhao HM,2013 | -0.95(-1.57,-0.32) | Yang XS,2007 |
